# The conceptualisation of vital communities related to ageing in place: a scoping review

**DOI:** 10.1007/s10433-021-00622-w

**Published:** 2021-05-10

**Authors:** Katinka E. Pani-Harreman, Joop M. A. van Duren, Gertrudis I. J. M. Kempen, Gerrie J. J. W. Bours

**Affiliations:** 1 Department of Health Services Research, Maastricht University, Care and Public Health Research Institute, Maastricht, the Netherlands; 2 Research Centre for Facility Management, Zuyd University of Applied Sciences, Nieuw Eyckholt 300, 6419 DJ Heerlen, the Netherlands; 3 Research Centre for Community Care, Zuyd University of Applied Sciences, Heerlen, the Netherlands

**Keywords:** Community networks, Vital communities, Community, Older people, Aged, Scoping review

## Abstract

Older people today are more likely to age in their own private living environment. However, many face declining health and/or other issues that affect their ability to live independently and necessitate additional support. Such support can be provided by formal networks, but a considerable part can also be offered by informal networks of older people themselves. Going beyond these networks, older people can additionally and perhaps even more substantially benefit from *vital communities*. Nevertheless, even though this term is increasingly common in the literature, its meaning remains indistinct. A more thorough understanding of this concept might provide valuable knowledge that health care professionals, researchers and community workers can use to offer meaningful and effective support. The purpose of this paper is to draw on existing empirical research on vital communities to build knowledge of the different descriptions and dimensions of the concept. Arksey and O’Malley’s scoping review methodology was adopted. Our search, conducted on 23 March 2020 and updated on 06 January 2021, yielded 4433 articles, of which six articles were included in the scoping review. We deduced that the conceptualisation of a vital community is based on three dimensions: the aim of a vital community, the processes behind a vital community and the typical characteristics of a vital community. None of the selected studies have mapped all three dimensions. Nevertheless, we assume that understanding all three matters when vital communities aim to contribute to the quality of life of people ageing in place.

## Introduction

The world’s population is ageing rapidly. This is apparent both in terms of apparent numbers and growing proportions of older people within different countries’ populations across the world (World Health Organization (WHO) 2015). In Europe, the percentage of people aged 65 and older is increasing at an unprecedented rate and is expected to account on average for over 30% of the continent’s population by 2060 (European commission (EU) 2015). Today, older people age in their own private living environment (ageing in place) and for longer compared to in past decades. On the one hand, this development is influenced by changes in policy and regulations and, on the other, by older people’s wish to keep their independence (Machielse 2016; Thomas and Blanchard 2009; Vermij 2016). Grimmer et al. (2015) stated that ‘ageing in place’ is mostly about the opportunity for older people to remain in their own home for as long as possible, without having to move to a long-term care facility. Horner and Boldy (2008) defined ‘ageing in place’ as a ‘positive approach to meeting the needs of the older person, supporting them to live independently, or with some assistance, for as long as possible’. Such a desire for continued independence probably owes to the fact that independent living helps older people to maintain a sense of self-reliance, self-management and self-esteem (Milligan 2012). However, many older people face declining health, limitations in their functioning and/or other issues that affect their ability to live independently. Thus, older people who face such challenges but wish to continue living at home may require additional support (Machielse 2016). Golant (2015) describes the problems that ageing in place can cause when people reach the last stages of old age. They often need caregivers, and in many cases, the people who give the care are family members. Formal networks can partly provide this support, but the (informal) social networks of older people themselves may also make an important contribution. Formal support, provided by professionals, mainly consists of the infrastructure, facilities and services available to the older people in question. These services include public transportation, grocery stores, pharmacies, meal services and personal care (Dobner et al. 2016). However, a lack of amenities (e.g. grocery stores, pharmacies) and public transport options present significant hurdles to ‘ageing in place’, especially in disadvantaged neighbourhoods.

Strengthened and fostered community support and social cohesion may mitigate infrastructural deficiencies. The providers of informal support for older people in need include family members, neighbours, friends and the community in general. Such informal support consists of tasks such as light housework, meal preparation, shopping, finances and transportation (Wilkinson-Meyers et al. 2014). Focusing on informal community support and informal networks among neighbours, Dobner et al. (2016) have highlighted how informal networks (friends, neighbours, community) may become even more vital for older adults living far away from family members. However, for successful day-to-day support, the majority of one’s social network should be in relatively close proximity, and neighbours should be willing to interact and check up on each other. Moreover, even if both of these aspects are present, a social network will not function well if there is limited engagement or willingness and ability to work together among the members of the social network (Nocon and Pearson 2000).

Going beyond (informal) social networks, older people may also—and perhaps even more substantially—benefit from *vital communities.* This concept has its roots in the early community psychology literature from the 1960s and 1970s. At that time, community vitality was defined as the ability of communities to collectively solve problems (Scott 2010). Although one may assume that such communities share the characteristics of social networks, in reality they may be made up of numerous people and explicit common goals or shared interests. Apart from these general characteristics, different notions seem to exist about how vital communities are described and what kinds of vital communities exist. According to Dale et al. (2010), definitions of vital communities are mainly found in reports published by a variety of organisations, yet few scientific publications exist on their characteristics and the use of the concept. In a primarily theoretical paper, Scott (2010) asserts that vital communities are characterised by strong, active and inclusive relationships between residents, the private sector, the public sector and social organisations. Together, these stakeholders promote individual and collective well-being. Vital communities are depicted here as communities that are able to cultivate relationships and thereby create an environment where citizens can adapt and thrive, enjoying improved well-being in a changing world (Scott 2010).

A more thorough understanding of vital communities might help create valuable insights for health care professionals, researchers and community workers to offer meaningful and effective support of complementary interest for older people’s quality of life. The purpose of this paper is to draw on existing empirical research into vital communities to build knowledge about the different descriptions and dimensions of the concept to contribute to the formulation of better policies and the development of better practice in serving older adults.

## Methods

A scoping review is particularly useful for comprehensively and systematically mapping the literature and identifying the key concepts, theories, evidence and/or research gaps that exist in a broadly covered topic (Arksey and O'Malley 2005). It also allows for an analysis of papers that describe studies with diverse designs. Given that both of these features of scoping reviews fit the purpose of our study, which aimed to provide an overview of the different descriptions and dimensions of the concept of vital communities, we adopted this method to answer our research question. More specifically, we applied the scoping review methodology outlined by Arksey and O’Malley, which details an approach consisting of five stages: (a) identifying the research question, (b) identifying relevant studies, (c) selecting studies (d) charting the data and (e) collating, summarising and reporting the results.

### Identifying the research question

In order to provide an overview of the different descriptions and dimensions of the concept of vital communities, we defined the following research question: “What descriptions, dimensions and characteristics of the concept of vital communities have been distinguished in the scientific literature?”.

### Identifying relevant studies

The main goal of the second stage was to create an overview of sources discussing vital communities (in this case) to an extent that is relevant for analysis. Prior to this study, the authors conducted a scoping review of the concept of ageing in place. During that study, they were particularly looking for definitions, key themes and aspects of the concept of ageing in place (Pani-Harreman et al. 2020). For the current study, we were particularly focusing on the concept of vital communities. To achieve this goal, we conducted an exploratory literature study in books and articles (Dale et al. 2014; Deindl and Brandt 2017; Grigsby 2001; Hwang et al. 2008; Moulaert and Garon 2016). The literature study increased the authors’ familiarity with the literature and allowed them to operationalise the term *vital communities* into synonyms (e.g. “community participation”, “social environment”, “social participation”, “social marginalisation”, “social responsibility”) and linked search terms (e.g. “society”, “social welfare”, “social cohesion”, “neighbourhood”, “social networks”, “community”, “vitality”). The authors used these synonyms and linked search terms to develop a number of terms (e.g. “community networks”, “community network”, “vital community”, “vital communities”, “community health network”, “community health networks”, “community care network”, “community care networks”) that could be utilised to conduct searches within relevant databases and search engines: (a) PubMed, (b) PsychInfo, (c) CINAHL and (d) Scopus. The next step in this stage was to identify the most useful combination of search terms for each database. Table 1 presents the full electronic search strategy for the PubMed database, such that it could be repeated (Tricco et al. 2018). This task was carried out by two scientific reviewers (author KEPH and researcher SdG) independently. The two reviewers subsequently discussed their findings until they reached a consensus on one combination of search terms for each database. In this way, a different combination of search terms was identified for each database.

**Table 1:** Steps and detailed search terms used in the PubMed search

Step 1 search term	Step 2 MESH term PubMed	Step 3 Entry terms PubMed	Step 4 free text words	Step 5 search strategy
Vital community	Community network	Community network Community health networks Community care networks	Community network(s) Community health network(s) Community care network(s) Vital community Community participation	Vital community[tiab] OR Vital communities[tiab]
				Community Networks"[Mesh] OR vital community[tiab]
				“vital community” OR “vital communities” OR”community participation”
				Community Network[Mesh] OR vital community[tiab] OR community networks[tiab] OR community network[tiab] OR community health network[tiab] OR community health networks[tiab]
				Community Network[Mesh] OR vital community[tiab] OR vital communities[tiab] OR community networks[tiab] OR community network[tiab] OR community care network[tiab] OR community care networks[tiab]
				Community Network[Mesh] OR vital community[tiab] OR vital communities[tiab] OR community networks[tiab] OR community network[tiab] OR community health network[tiab] OR community health networks[tiab] OR community care network[tiab] OR community care networks[tiab]

Table 2 shows the combination of search terms used for each database as well as the number of hits that resulted from the search. Additionally, the search engine Google Scholar was used to optimise the results of the electronic database searches and to improve the reliability of the search strategy (Bramer et al. 2017). We conducted a search in March 2020 and updated this search in January 2021, with no restrictions on the date of publication. Reference lists of the included articles were also screened to identify additional key studies.

**Table 2:** Search terms and search strategy scoping review vital communities

Database	Search strategy	Hits
PubMed	“community networks” [mesh] OR “vital community” OR “vital communities” OR “community networks” OR “community network” OR “community health network” OR “community health networks” OR “community care network” OR “community care networks”	2510
CINAHL	(MH “community networks”) OR “vital community” OR “vital communities” OR “community health network” OR “community health networks” OR “community care network” OR “community care networks”	370
PsychInfo	“community networks” OR “community network” OR “vital community” OR “vital communities” OR “community health network” OR “community health networks” OR “community care network” OR “community care networks”	536
Scopus	“community networks” OR “community network” OR “vital community” OR “vital communities” OR “community health network” OR “community health networks” OR “community care network” OR “community care networks”	485
Google Scholar	(“vital community” OR “vital communities”) AND (“older people”)	532
Total		4433

### Selecting studies

The third stage was aimed at facilitating the extraction and analysis of data from relevant papers by selecting from the articles retrieved in the identification stage. Studies were eligible if they: (1) described the concept, (2) described a definition, (3) and/or described the characterisation of a vital community, (4) were original research articles (quantitative and/or qualitative empirical studies, systematic reviews, meta-analyses, meta-syntheses and scoping reviews) and (5) were written in English, German or Dutch.

This study selection process consisted of assessing the articles in three steps, first by focusing on the title, then the abstract and then the full text of each article. The reviewers divided the studies into one of three categories (relevant, irrelevant or doubtful) for each step of the process. To validate the selection procedure, the eligibility criteria were independently checked by two reviewers (author KEPH and researcher SdG) for consistency. This assessment was first made by checking the title of each article and then by reading its abstract. After screening the titles and abstracts, the articles that were deemed eligible were obtained as full texts and then further scanned for eligibility. A logbook was used to record the reasons for excluding studies based on their full texts. The studies that remained after the third stage of selection were considered relevant for this scoping review. If the two reviewers did not agree on the relevance of a particular study, a third reviewer (author GJJWB) was asked to determine its suitability. To facilitate the selection process, Endnote X9 was used to import the title, author(s), date of publication, journal of publication, abstract and full text (if available) of each article resulting from the searches. This information was used to keep track of the selection process by sorting articles along the lines of inclusion and exclusion. A logbook was used to record the number of articles resulting from each phase of the selection process and Endnote X9.

### Charting the data

The fourth stage of the scoping review involved charting key items of information obtained from the papers being reviewed. Charting is a technique by which qualitative data are synthesised and interpreted via sifting and sorting material according to key issues and themes (Arksey and O'Malley 2005). To facilitate the data selection, the authors agreed to use a chart on which they noted all information that was considered useful. Two reviewers (authors KEPH and GJJWB) independently charted the data from each article, discussed the results and continuously updated the data chart in an iterative process. This data chart contained the following descriptive variables: author(s), year of publication, country of origin, research aim, research question, study population, sample size, research methodology, descriptions given by the author(s) of vital communities and key findings.

### Collating, summarising and reporting the results

The fifth stage of a scoping review involves collating, summarising and reporting the results (Arksey and O'Malley 2005). Focusing on the descriptions and the characteristics of vital communities, we applied a qualitative content analysis in which we used an open, axial and selective coding method (Levac et al. 2010). The data from the articles were inductively coded in Excel. With open coding, labels are linked to the fragments from stage four (e.g. charting the data). These labels summarise the core of the fragments. The coding scheme is refined by clustering codes together to make categories (axial coding) during the conceptualisation of similarities and differences in the codes. Conceptual saturation is reached when no new categories are generated from the open codes. The categories are then examined for their relationships to each other (selective coding) to add overarching categories.

## Findings

### Study characteristics

**Fig. 1:**
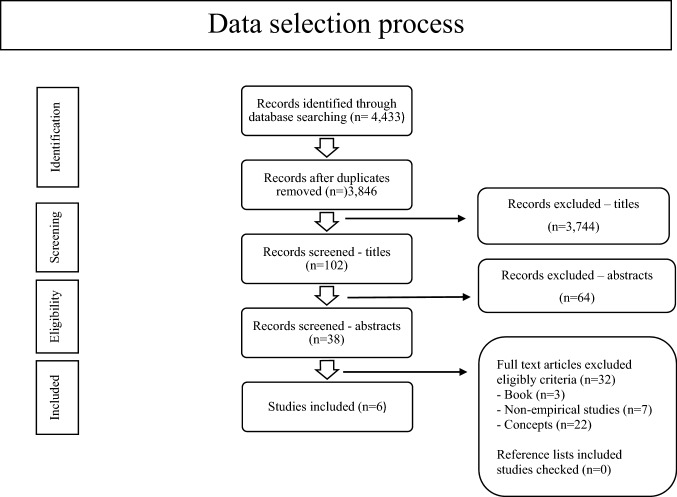
Data selection scoping review vital communities

The four electronic databases and the search engine Google Scholar were searched on 23 March 2020 and updated on 6 January 2021 with no restrictions on publication dates. Based on the search, 4433 articles concerning vital communities were identified. Next, 587 duplicate articles were removed. The titles of the remaining articles were then independently reviewed and 3744 articles were excluded from the study based on their titles. Out of the remaining 102 articles, independent screenings of the abstracts led to 38 articles still being considered relevant. A final assessment of these articles, this time taking their full texts into account, left a final number of six relevant studies for the scoping review. An overview of the data selection process is shown in Fig. 1.

The selected articles were published between 2008 and 2020 and focused on the following different geographical locations: the United States (*n* = 4), Canada (*n* = 1) and Europe (*n* = 1). The studies centred on a research population of communities, key informants (e.g. federal and state government, philanthropy, national associations, regional/local health and ageing services organisations), outreach workers, residents and members of a service exchange programme. Different methodologies were used in the six selected studies: interviews (*n* = 2); survey (*n* = 1); secondary data analysis (*n* = 1); and meta-case study (*n* = 2). The study characteristics of the selected articles are shown in Table 3 (Tables 3 and 4).

**Table 3:** Study characteristics included research papers

Author(s) (Country, year)	Study population (sample size)	Research method	Research aim and/or question	Key findings
Berman, Murphy-Berman and Melton (2008, United States)	Outreach workers Community members Age: 20 or older Gender (male): 36% Gender (female): 64% (n = 676)	Data analyses of the biweekly reports	To describe the work of the outreach staff in strong communities and to evaluate whether it conformed to the principles on which the initiative was based, as described in the introduction to this issue of *Family & Community Health*. Second, to provide future innovators with concrete ideas concerning whom they might approach and what kinds of activities they might suggest when they attempt the difficult task of transforming a community	The results showed that strong communities have indeed penetrated into the target communities in diverse ways, engaging people of disparate backgrounds. The first years of the initiative showed the feasibility of engaging primary community institutions in a broad-based effort to enhance children’s safety in their homes and the community at large. They indicated the importance of community gatekeepers and of seemingly “natural” but actually constructed events and groups in facilitating such efforts 64% of the individuals named in the biweeklies were women. The “typical” person who is central in an activity in Strong Communities is a woman, white, and between the ages of 30 and 49
Foster-Fishman, Pierce, and Van Egeren (2009, United States)	Residents living in the seven poor neighbourhoods involved in YWC Age: 18 or older Gender (male): 23% Gender (female): 77% *(n* = 205)	Survey	To examine the factors associated with citizen participation levels in resident leaders and followers in seven low-income neighbourhoods in one community	Overall, the findings suggested that different factors facilitate participation in leaders and followers. Leaders are more likely to actively participate in neighbourhood and community affairs if they perceive themselves as having the skills needed to organise others and make change happen. Whereas perceived skill levels also matter for followers, these residents are strongly influenced by the norms for activism within their neighbourhood. These norms mediate the impact of neighbourhood readiness and the capacity for change on citizen participation levels
Letcher and Perlow (2009, United States)	Members of a service exchange programme in an urban community Age: 23–84 Gender (male): 28% Gender (female): 72% (*n* = 211)	In-depth interviews	To explore how diverse participants engage in a supportive network and proposes a theoretic model of community building for health promotion	Four primary themes related to participation in the service exchange programme were identified: (1) motivation for participation; (2) service exchange, or reciprocity, as vital to the programme, with distinct benefits in a heterogeneous group; (3) occurrence of personal and community growth; and (4) health promotion and improved well-being. A model of how participation in the service exchange leads to community building is presented
Altpeter, Schneider and Whitelaw (2014, United States)	Key informants (e.g. federal and state government, philanthropy, national associations, regional/local health and ageing services organisations) Age: not mentioned Gender: not mentioned (*n* = 11)	Interviews	To learn how ageing and health collaborations created strategic partnerships to foster multisector systems change and pursue long-term goals and near-term activities to sustain and expand evidence-based health programming	Four creative strategies emerged across sites as contributing to the growth and sustainability of evidence-based health programming including engagement of non-traditional partners, development of new relationships with health care, building of innovative systems of structures and tools, and systematically working with vulnerable populations
Dale, Ling and Newman (2010, Canada)	Canadian communities Age: not mentioned Gender: not mentioned (*n* = 35)	Mixed-methods Meta-case analysis	To outline how community vitality acts as a cornerstone of sustainable development and suggests some courses for future research	The analysis of the thirty-five case study communities revealed common features that can be deemed characteristic of community vitality: Community openness and trust Connection with people and place Continuity and stability of funding and leadership Perturbation Diversity
Machielse, Vaart van der (2020, The Netherlands)	Residents of 10 selected complexes in the Netherlands Age: 55 or older Gender (female): 76% Gender (male) not mentioned (*n* = 405)	Mixed-methods Meta-case analysis	To explore the possibilities of residents in low-income housing complexes to improve the social quality in their complexes and to get insight into the need for professional support	Results showed that the self-organising capacity of the residents is limited due to a lack of knowledge and organisational skills, and health problems. Improving social quality requires permanent attention from facilitating professionals, who guide the process and ensure continuity

**Table 4:** Coding tree, dimensions of vital communities

Selective coding dimensions of vital communities	Axial coding	Open coding
The aims of vital communities	To create beneficial partnerships	Creating mutually beneficial partnerships (Altpeter et al. 2014)
	To support programme expansion and cultural change	Building system of structure and tools, supporting programme expansion and cultural change (Altpeter et al. (2014)
	To reinforce the sense of belonging	Improving quality of life and sense of belonging (Letcher and Perlow 2009)
	To reinforce the quality of life	Improving quality of life and sense of belonging (Letcher and Perlow 2009)
Mechanisms behind a vital community	Strategies	Implementing creative strategies for growth and sustainability (Altpeter, Schneider et al. 2014)
	Creativity and innovation	Stimulating community vitality through innovation and creativity (Dale et al. 2010)
	Community- and service exchange	Creating vital communities through community and service exchange, leading to relationships that in turn create community (Letcher and Perlow 2009)
	Partnership	Pursuing innovation and creativity (thus vital communities) by secure and stable leadership and particularly private/public partnerships (Dale et al. 2010)
	Active participation	Stimulating active participants and volunteers (Berman, Murphy-Berman et al. 2008)
	Community capacity	Stimulating a sense of community by improving community capacity (relationship between neighbourhood readiness, capacity and citizen participation) (Foster-Fishman, Pierce et al. 2009) The self-organising capacity of the residents is limited due to a lack of knowledge and organisational skills (Machielse, Van der Vaart 2020)
	Community skills	Stimulating (citizen) participation by leadership, neighbourhood conditions and community skills (Foster-Fishman, Pierce et al. 2009)
	Leadership and funding	Continuity and stability of leadership and funding (Dale et al. 2010)
	Perturbation	Maintaining the vitality of communities by perturbation Perturbation stimulates innovation and creativity, leading to community action and vitality (Dale et al. 2010)
	External change	Enhancing vitality by external change (Dale et al. 2010)
Typical characteristics of a vital community	Personal and collective growth	Vital communities create environments of both personal and collective growth, fuelled by member engagement (Letcher and Perlow 2009)
	Openness and trust	Vital communities are characterised by community openness, community trust and communication (Dale et al. 2010)
	Cohesion	Community vitality is related to the degree of community cohesion (Dale et al. 2010)
	Shared vision	Vital communities are characterised by a shared vision (Dale et al. 2010)
	Connection to people and place	Vital communities are characterised by connection to place (generally not the built environment, but the people and the social capital in the specific location) (Dale et al. 2010)
	Engagement, involvement and empowerment	Vital communities are characterised by the engagement of members (Berman et al. 2008), the involvement of various demographic groups (Berman et al. 2008), the involvement of community sectors (Berman et al. 2008) and by the engagement and empowerment of vulnerable populations (Altpeter et al. 2014)
	Resilience	The heuristics of community vitality are resilience, innovation and adaption (Dale et al. 2010)
	Sustainment, growth and advocacy	Vital communities are characterised by the needs and opportunities for sustainment, growth and advocacy (Altpeter et al. 2014)
	Diversity	Diversity is a basic component of community vitality (Dale et al. 2010)
	New collaborations	Vital communities are promoting new collaborations with broader missions (Altpeter et al. 2014)
	Adaption	The heuristics of community vitality are resilience, innovation and adaption (Dale et al. 2010) Vital communities support organic development and adaption (Altpeter et al. 2014)
	Benefits from participation	The benefits of participation in community exchange range from access to affordable services, to meaningful relationships, to community mobilisation (Letcher and Perlow 2009)

The results of the data analysis yielded only one definition of vital communities. We deduced that the conceptualisation of a vital community is based on three dimensions: the aim of a vital community, the processes behind a vital community and the typical characteristics of a vital community.

### Definitions of vital communities

The results of the data analysis yielded only one definition of vital communities, by Dale et al. (2010), as follows:

A vital community is one that can thrive in the face of change. It is a place that can remain at its core a functional community without loss to ecological, social and economic capitals in the long run, whatever occurs as a result of exogenous changes beyond its control. And perhaps more importantly, it is a place where human systems work with rather than against natural systems and processes (p. 217, introduction).

### Dimensions of vital communities

Three main dimensions of vital communities were identified: (a) the aim of a vital community (the ‘why’), (b) the mechanisms behind a vital community (the ‘how’) and (c) the typical characteristics of a vital community (the ‘what’). The first dimension describes the reasons for existence. The second dimension represents the processes behind a vital community. Finally, the third dimension represents the key characteristics of vital communities, in other words, the characteristics that make a community a vital community. The structure of the dimensions resulting from the data analysis is shown in Table 3.

### The aim of a vital community

Two of the six studies included described the aim of a vital community: Altpeter et al. (2014) discussed vital communities as aiming to create beneficial partnerships in order to support programme expansion and cultural change, while Letcher and Perlow (2009) described these communities as aiming to reinforce their members’ sense of belonging and quality of life. According to Altpeter et al. (2014), vital communities seek to create mutually beneficial partnerships with health care organisations by building strong partnerships with community care partners. The purpose of vital communities is to build systems of structures and tools to support programme expansion to make permanent impacts. Letcher and Perlow (2009) found that vital communities reinforce the improved well-being of their members. They also identified members’ sense of belonging to a community and improved perceived quality of life. Moreover, as members developed a supportive network, they enhanced their resilience in times of stress.

### Mechanisms behind a vital community

Five of the six studies included described the mechanisms behind a vital community that influence its vitality. The mechanisms found were the following: (a) creative strategies, creativity and innovation, (b) partnership, community and service exchange and active participation, (c) community capacity, community skills, stable leadership and funding and (d) perturbation and external change.

#### Creative strategies, creativity and innovation

Developing and implementing creative strategies ensure the growth and sustainability of a vital community. According to Altpeter et al. (2014), creative strategies facilitate the development of mutually beneficial partnerships and service exchange, empowering vulnerable people and building systems of structures and tools to support programme expansion and cultural change in order to make permanent impacts. Dale et al. (2010) identified creativity and innovation as two other mechanisms that stimulate the vitality of a community. For example, they highlighted evidence that minority opinions stimulate creativity and divergent thoughts, which can result in innovation during participation.

#### Partnership, community and service exchange and active participation

Dale et al. (2010) found that (particularly private and public) partnerships expand the public sphere to pursue innovation and creativity, leading to community vitality. Letcher and Perlow (2009) noted that community and service exchange lead to relationships with mutual benefits that in turn create a sense of community. The benefits of service exchange include inclusion, individuals taking on new roles, respect and appreciation for others and a network of friends. Exchange allows those who have been socially isolated or stigmatised to build relationships. Community members like having a network of partners who are willing to build such relationships. Indeed, they like having a team and working together to offer reciprocal instrumental and social support, without distinguishing those who give from those who need services. Exchange also encourages people to take on new roles. As members stretch themselves to honour the community’s expectation of reciprocity, they learn to respect others. Relationships emerge out of a network where everyone has opportunities to give and receive and to be recognised for their contributions. Service exchange allows members to get to know each other based on sharing. Members joining community exchange may be motivated instrumentally. Furthermore, some members perceive their current participation in a community as an investment in social capital or as an ‘insurance’ policy that gives them the confidence that help will be available to them in the future. Vital communities need highly active participants and a body of exceptional volunteers as kick-starters and endurance power (Berman et al. 2008). In Berman et al.’s study, the most active participants were not elected officials, corporate leaders or the individuals named to relevant citizen boards but rather those who could easily leverage the resources of a particular community institution and who were committed community servants.

#### Community capacity, community skills, stable leadership and funding

Vital communities create strong relations that promote community readiness and capacity. For example, Foster-Fishman et al (2009) found strong support for their hypothesis that activism norms would mediate the relationship between community readiness and capacity and citizen participation. Their study also showed that the organising skills of a vital community represent a strong direct predictor of citizen participation. According to a study of Machielse and van der Vaart (2020), the self-organising capacity of community members is limited due to a lack of knowledge and organisational skills and health problems. Other research indicated that vital communities need community leaders and gatekeepers to work in a complementary manner. For example, Dale et al. (2010) showed that vital communities require substantial organisational skills, these being crucial to participation. This study also showed that men in vital communities often provide long-term direction while women take care of day-to-day leadership. Stable and secure leadership is important and expands the public sphere to pursue innovation and creativity. In addition, Dale et al. (2010) demonstrated that stable and secure leadership is directly linked to community vitality. The continuity of funding was identified as a key element in protecting the leadership from the constant stress of fundraising and related burnout. This in turn allowed for the stability of leadership, as the core group was maintained. Such stability is often severely lacking in civil society organisations, especially in grassroots and smaller groups.

#### Perturbation and external change

Dale et al. (2010) found that perturbation and stability are also important, as change inhibits vitality. They identified a possible link between the degree to which a community is stable and can respond to change on the one hand and its functional social diversity on the other. If perturbation occurs in such a way, that it maintains core stability, then it actually stimulates vitality. In other words, perturbation may promote the resilience of a community: the community is stable and able to respond adequately to perturbation. It seems clear that perturbation is needed to stimulate action and, in some cases, vitality. Changes are necessary, with the key element being to build redundancy at the local level alongside resilience as a buffer, especially for coping with exogenous shocks so that change does not prove catastrophic. This reinforces the importance of both variables for sustainable community development, as change helps enhance vitality, assuming that vitality and sustainable development are linked (Dale et al. 2010).

### Typical characteristics of a vital community

The third dimension of a vital community is its typical characteristics, defined as typical or noteworthy qualities. According to the outcomes of the previous studies, such characteristics make a community a vital community. We have arranged these characteristics on three levels: (a) individual, (b) collective, that is, all members together and (c) the vital community as an entity.

#### Individual characteristics

We can note the following individual characteristics of community members: attachment to place, engagement, involvement and empowerment. Vital communities are based upon connections between people and attachment to place. A sense of the meaning of the place within the community stimulates community attitudes and values. Attachment to place is manifested less in terms of the built environment and more with the people and the social capital that exist in the specific location, developed through networks of empowerment (Dale et al. 2010). Engaging, involving and empowering individuals provide the essence of a vital community. According to Berman et al. (2008) participant involvement is a key characteristic. In a project that seeks to facilitate community change, it is important to understand the quantity and the type of people involved. Moreover, Altpeter et al. (2014) argued that through community initiatives, a vital community systematically engages and empowers vulnerable people to address older adult health and well-being.

#### Collective characteristics

The identified collective characteristics of a vital community are community cohesion, resilience and diversity. A vital community is characterised by cohesion. According to Dale et al. (2010), it is plausible that community vitality is related to the degree of community cohesion and that there may be an integral relationship between adaptive governance, stability and community vitality. The results of Dale et al.’s study additionally distinguish three heuristics of community vitality: resilience, innovation and adaption. Resilience is a function of the social networks that form part of a community and can be measured by indicators such as variability of income, stability of livelihoods, wealth distribution, demographic change and agency. Vital communities require sustainment, growth and advocacy (Altpeter et al. 2014). Diversity is one of the basic characteristics of community vitality and ensures the sustainable development of a vital community (Dale et al. 2010). It is a basic characteristic because of complementarity in the group: relationships within the network create an environment of both personal and collective growth that is fuelled by member engagement. In Letcher and Perlow’s (2009) study, several respondents described the emerging leaders who were engaged in activities that would strengthen the group as a whole (e.g. recruiting new members, developing programmes, offering classes, organising events). The entire network can become stronger as more members begin to engage in complex tasks together, ranging from organising meals and leisure activities to gathering a community of help when required by people. Community exchange thus establishes a powerful mechanism for social engagement or, as one member explained, a way of “having a stake in the community” (Letcher 2009, p. 296).

#### Vital communities as an entity

We can also note the characteristics of a vital community as an entity. These include having a collective or shared vision and community openness and trust (Dale et al. 2010). Having a collective vision brings newcomers and the core community together, resulting in increased community vitality. The second typical characteristic of a vital community as an entity is community openness and trust. In terms of vitality, Dale et al. (2010) showed that communities are stronger and more sustainable when there is community openness and trust. Community openness also enables and facilitates transdisciplinary cooperation between community members. According to Altpeter et al. (2014), vital communities create new collaborations between broader target groups and broader missions. In their study, sites developed new collaborations extending beyond their established ageing service partners. Furthermore, in Letcher and Perlow’s (2009) study, the community members described the benefits of participating in community exchange, ranging from having access to affordable services to meaningful relationships and, finally, to community mobilisation. Indeed, members can help reduce the barriers to care by providing services such as transportation to medical appointments and respite care for families.

**Fig. 2:**
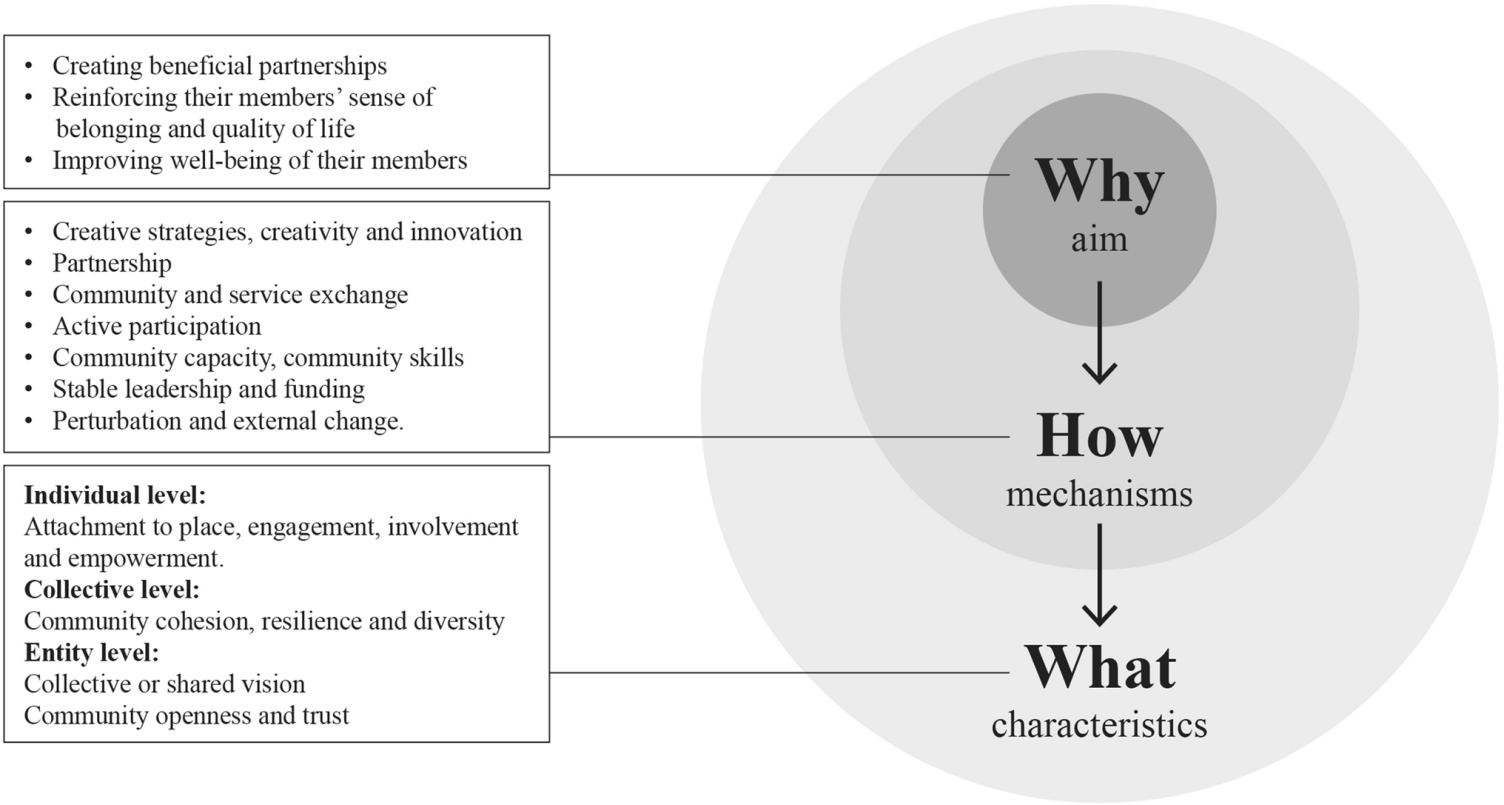
.

Given the complex and multidimensional nature of the concept of vital communities, a visual representation of the narrative is given in Fig. 2.

## Discussion

This scoping review was performed to gain greater insights into the different descriptions, dimensions and characteristics of the concept of vital communities, based on previous empirical research. The study demonstrates one definition and three dimensions that are largely congruent with the concepts and meanings of vital communities. Specifically, we have identified three dimensions of vital communities: (a) the aim of a vital community, (b) the mechanisms behind a vital community and (c) the typical characteristics of a vital community.

Altpeter et al. (2014) discussed vital communities as aiming to create beneficial partnerships in order to support programme expansion and cultural change by creating mutually beneficial partnerships with health care organisations, by building strong partnerships with community care partners. This aim differs from the aim of a Senior Friendly Community (SFC), framed by Schichel (2020) as focussing on older people’s public health, well-being and quality of life. In addition, the author’s opinion is that in a vital community the mutual exchange of giving and receiving support is key, while in a SFC one-way traffic (the community and environment being senior friendly) is dominantly present. However, the mechanisms, we found within communities that influence the vitality of these communities in order to achieve their shared goals, are remarkably comparable. The essence of these mechanisms exists in both variants of: (a) increased use of local skills and knowledge; (b) strong (mutual) relationships and communication; (c) taking the initiative and showing responsibility and adaptability; (d) the existence of sustainable, healthy ecosystems; and (e) varied and healthy economies (Flora 1998). The findings of these reviews and the findings of Flora (1998) contradict to the more practical and critical factors, stated by the MetLife Mature Market Institute (2014). According to this institute, most important critical factors are accessible and affordable housing, safe neighbourhoods, the presence of healthcare (programs), home and community-based caregiving support services, retail outlets, programs and organisations that promote social activities and intergenerational contact, senior transportation programs and walkable neighbourhoods. In summary, a vital community focuses more on mutual relations and support, while SFC focuses more on the tangible aspects. It would be interesting for future research to find out if, how and to what extent these two concepts can reinforce each other. Additionally, the structure of a vital community seems to be of minor importance, because our results have demonstrated, no typical characteristics of a vital community were found, with regard to the structure. Although one may assume that vital communities share the characteristics of social networks, in reality they may be made up of numerous people and explicit common goals or shared interests. It would be of interest to investigate the similarities and differences between vital communities and social networks in future research.

We may highlight Dale et al.’s (2010) definition of a vital community as a community that can thrive in the face of change. Dale et al. (2010) assume that a vital community is a physical place. However, today one can also question whether a vital community might exist virtually. The current Covid-19 pandemic could be an interesting external threat of a vital community. Perturbation is listed as a mechanism influencing the vitality of vital communities, and it would be of interest at some point in the future to assess what impact the pandemic has on the sustainability and resilience of vital communities.

### Strengths and limitations

This scoping review has several strengths. First, we used a comprehensive search strategy across multiple databases and a search engine with no date restrictions. This minimised the risk of missing scientific studies about vital communities. Second, to enhance trustworthiness, the process of selecting studies and extracting charting data was done independently by two reviewers (Levac et al. 2010). Nevertheless, the search conducted for this study may have also been subject to certain limitations. First, in our search strategy, we used a combination of keywords, but a vital community is a broad concept encompassing varied terminology. It is possible that we missed studies using other terms with similar meanings. In an effort to limit the negative implications of this issue, we checked reference lists. Second, we limited our search to quantitative and qualitative empirical studies, systematic reviews, meta-analyses, meta-syntheses and scoping reviews. As a result, we may have missed some descriptions of vital communities. However, we were especially interested in the ways in which vital communities have been described in previous empirical studies. The six studies included are from Europe, Canada and the United States. We did not find information concerning vital communities in other regions. There may be information in the grey literature, or vital communities may exist under other terms.

## Conclusions and implications

For this study, we formulated the following research question: “What is the meaning of the concept of vital communities and what descriptions, dimensions and characteristics have been distinguished in the scientific literature?” The concept of vital communities is broad and has only been defined in one empirical study. The analysis of the six studies included here has shown that three dimensions can be distinguished: (a) the aim of a vital community (the ‘why’), (b) the mechanisms behind a vital community (the ‘how’) and (c) the typical characteristics of a vital community (the ‘what’). If we want to understand the importance of vital communities and incorporate them into society, we must focus on the aim, the mechanisms and the characteristics. Therefore, health care professionals, researchers and community workers may consider the following questions:

- What do community members want to achieve at the individual level or at the level of the community?
- Through which processes and underlying mechanisms can these goals be achieved, and which characteristics at the individual, group and entity levels should therefore be promoted or developed?

Consequently, further research into the relationship between the three dimensions of vital communities is recommended. In addition, it is recommended that (local) government, health care organisations, service providers, housing corporations, neighbourhood associations, community workers and other community stakeholders unite and seek opportunities for collaboration and cooperation. Further research is also recommended into the relationship between vital communities and ageing in place in order to ensure meaningful and effective support that can be of complementary interest for older people’s quality of life while ageing in place.
